# Supplementary material for: A Plasmodium yoelii HECT-like E3 ubiquitin ligase regulates parasite growth and virulence
Source: Nat Commun. 2017 Aug 9;8:223. doi: 10.1038/s41467-017-00267-3 (PMC5548792; doi:10.1038/s41467-017-00267-3)
Supplement: Supplementary file 3 — Supplementary information [file 41467_2017_267_MOESM3_ESM.pdf]

Supplementary Figures

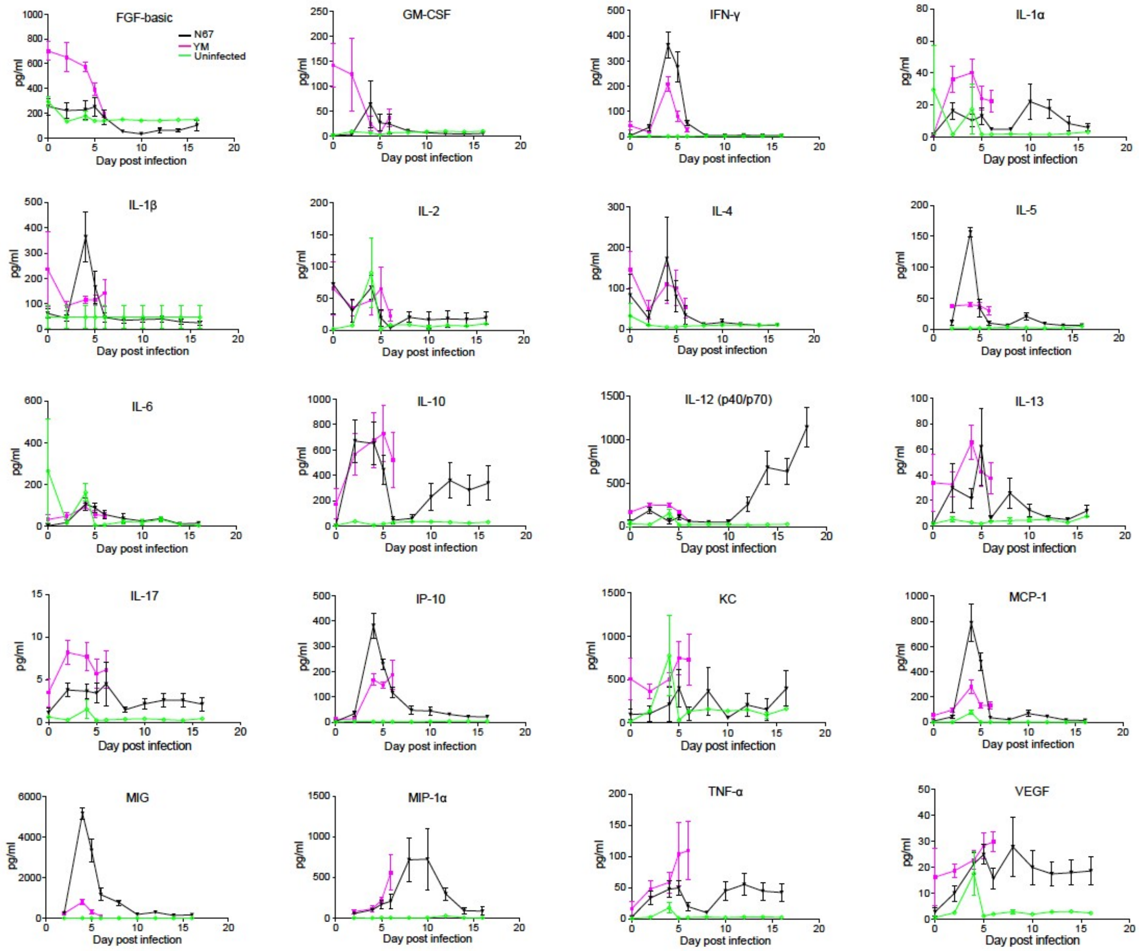

Supplementary Fig. 1. Cytokine and chemokine levels of mice infected with *Plasmodium yoelii nigeriensis* N67 and *P. y.yoelii* YM over time. Blood samples were collected at various days post infection, and cytokines and chemokines in the plasma were measured using a 20-plex Bead Array kit (Invitrogen). Blood samples from uninfected mice were also tested. Means and standard deviations were from 3-5 mice.

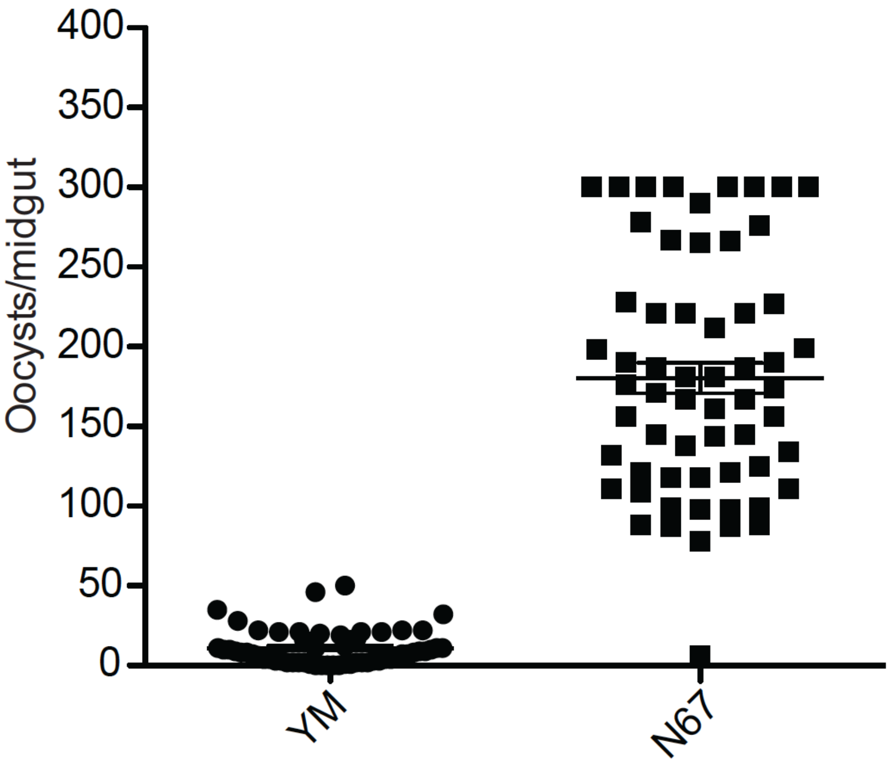

Supplementary Fig. 2. Oocyst counts from midguts of mosquitoes infected with *Plasmodium yoelii yoelii* YM or *P. y. nigeriensis* N67. Each dot or square represents oocyst counts from one mosquito midgut.

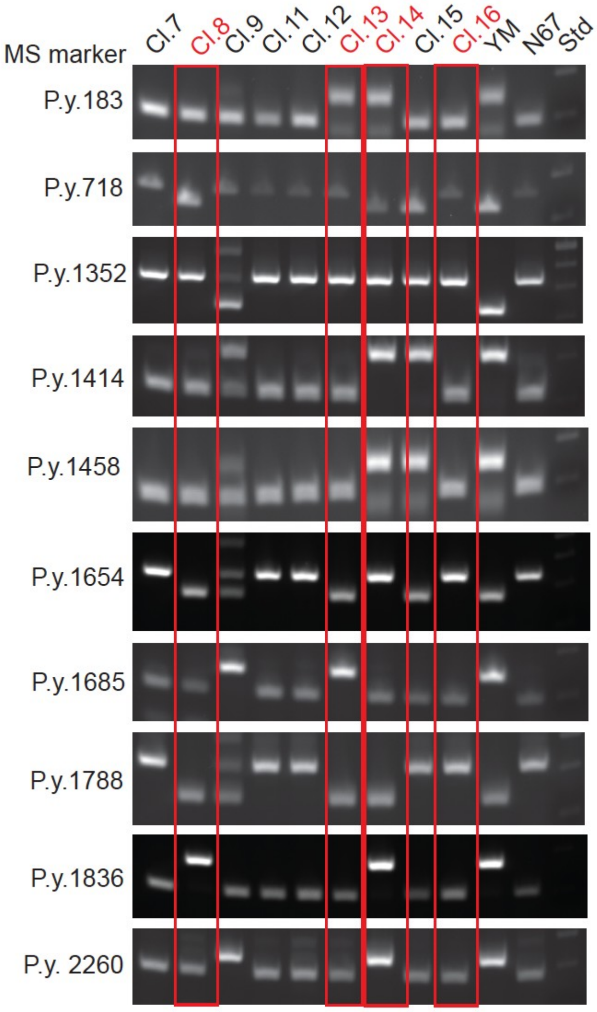

Supplementary Fig. 3. Agarose gels showing PCR products from ten microsatellites used for initial screening of parasite progeny of the *Plasmodium yoelii yoelii* YM and *P. y. nigeriensis* N67 crosses. Red color indicates potential independent progeny with unique marker combinations.

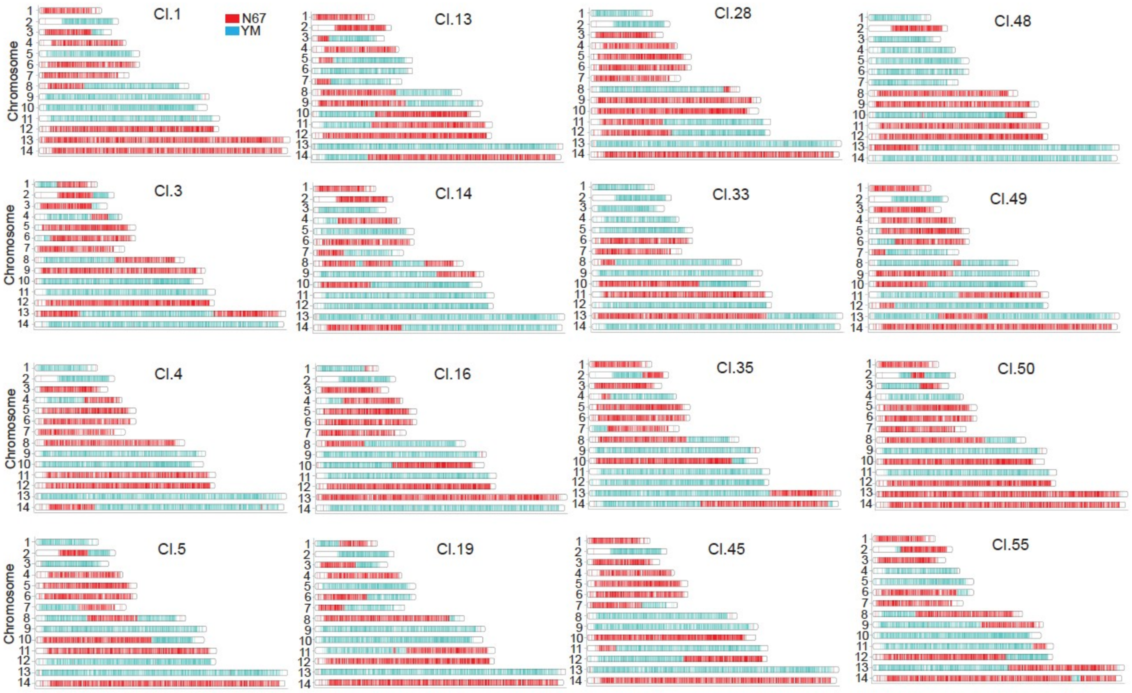
N67
YM
Chromosome
Cl.1
Cl.13
Cl.28
Cl.48
Cl.3
Cl.14
Cl.33
Cl.49
Cl.4
Cl.16
Cl.35
Cl.50
Cl.5
Cl.19
Cl.45
Cl.55

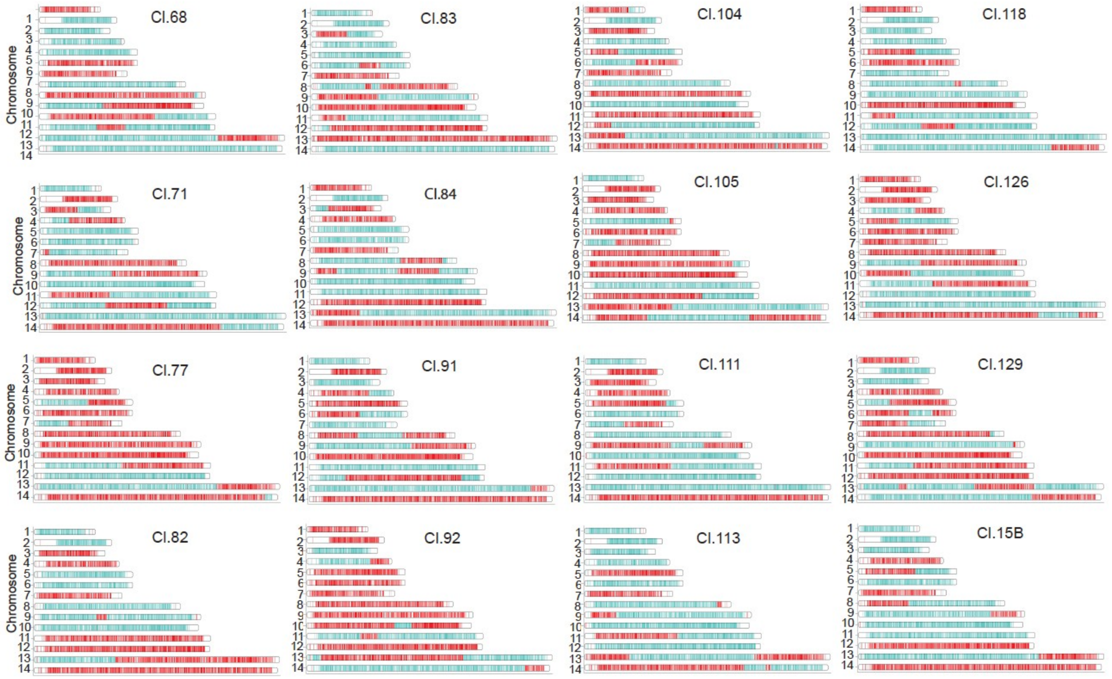
Chromosome
Cl.68
Cl.83
Cl.104
Cl.118
Cl.71
Cl.84
Cl.105
Cl.126
Cl.77
Cl.91
Cl.111
Cl.129
Cl.82
Cl.92
Cl.113
Cl.15B

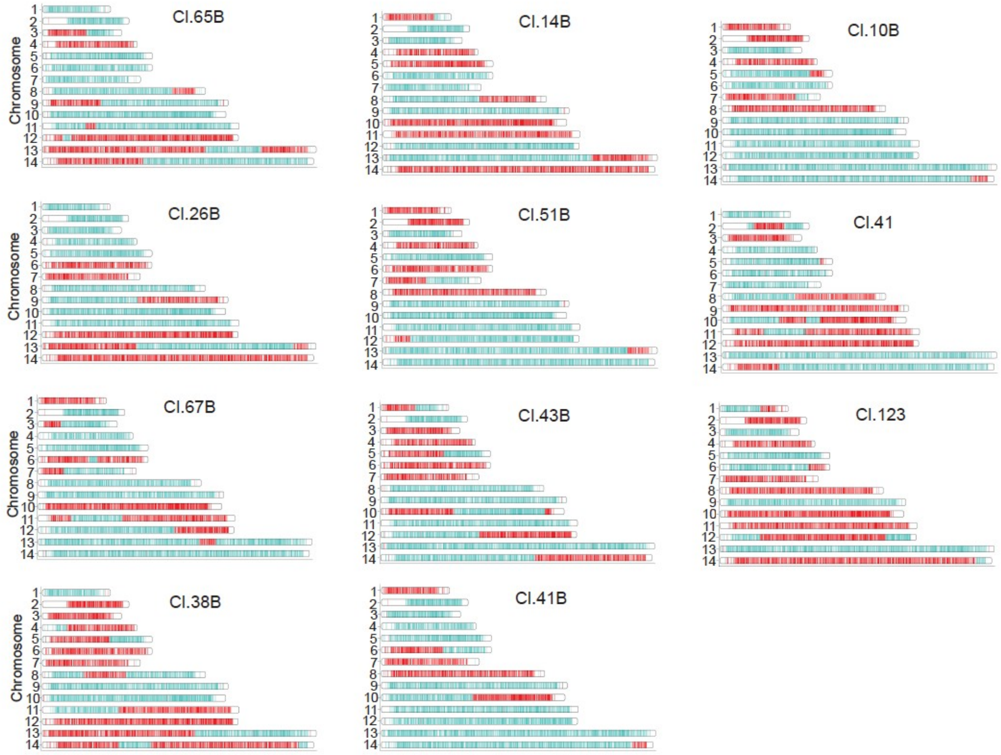

Supplementary Fig. 4. Graphic displays of genotypes of the parasite's 14 chromosomes from progeny of the YM × N67 cross. The progeny names are as indicated. Cyan, YM genotype of approximately 9,200 single nucleotide polymorphisms (SNPs); red, N67 genotype of SNPs. In each figure, chromosomes are arranged from chromosome 1 (top) to 14 (bottom).

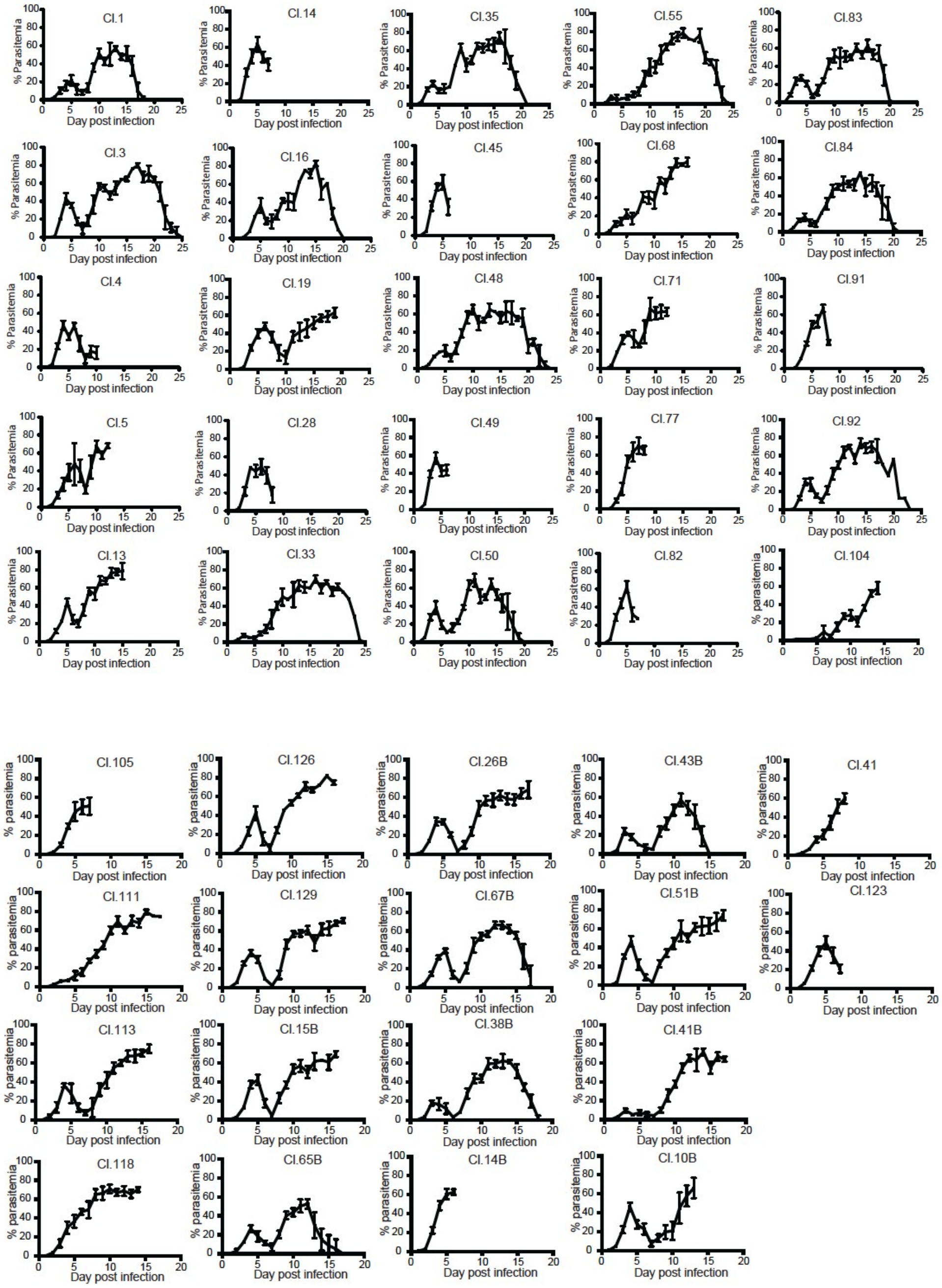

Supplementary Fig. 5. Parasitemia dynamics of the 43 progeny from the YM × N67 cross. Mice were infected with $1{\times}10^6$ parasites, and blood smears were made every other day from day 2 post infection until host death or clearance of parasites. The progeny names are as indicated. Mean parasitemia and standard deviations were calculated from 3-5 mice.

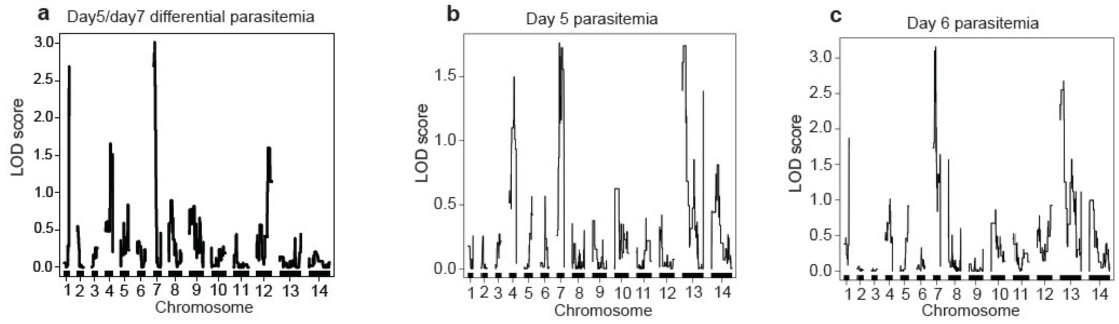

Supplementary Fig. 6. Plots of LOD scores (logarithm of the odds) for parasitemia from day 5-day 7 post infection with 1×10^6 parasites. Quantitative trait loci (QTL) analysis was performed as described [1,2]. (**a**) Difference between day 5 and day 7 parasitemia as phenotype. (**b**) Day 5 parasitemia as phenotype. (**c**) Day 6 parasitemia as phenotype.

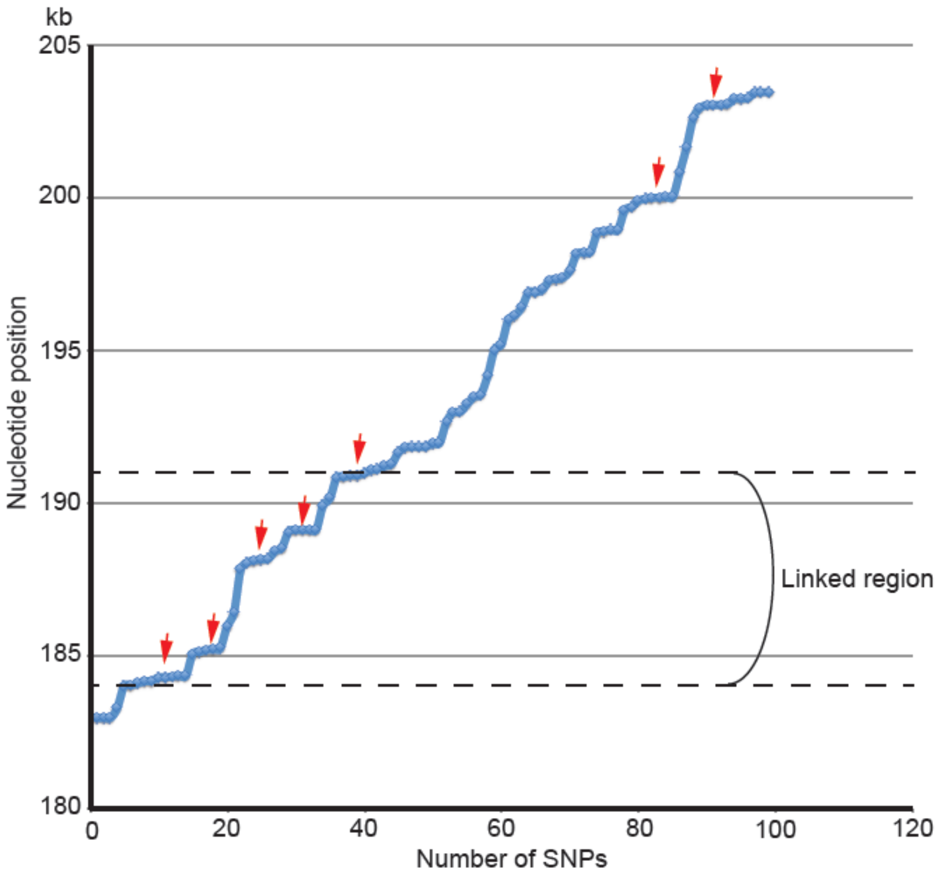

Supplementary Fig. 7. Nonsynonymous SNPs in the *Plasmodium yoelii* HECT E3 ubiquitin ligase gene. Nonsynonymous single nucleotide polymorphisms (SNPs) between *Plasmodium yoelii yoelii* YM and *P. y. nigeriensis* N67 were identified and plotted against their positions in the gene. The region between the dash-lines is the segment linked to the highest LOD (logarithm of odds) score. The red arrows point to highly polymorphic regions with clusters of SNPs. Note, there is no nonsynonymous SNPs at the 3' end of the gene spanning >1.5 kb (see Supplementary table 4).

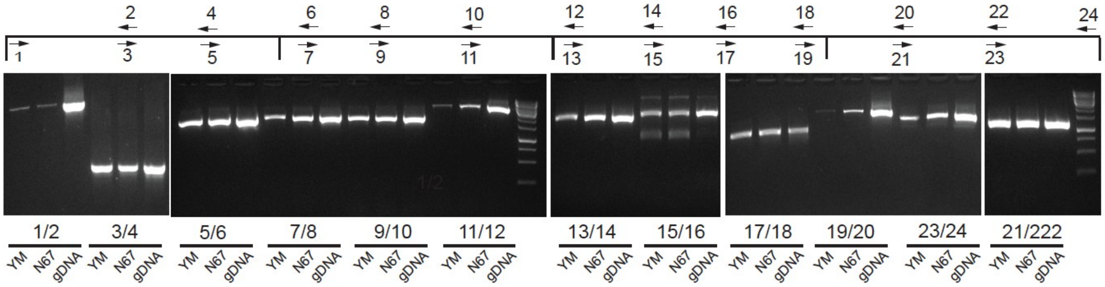

Supplementary Fig. 8. Tiling of RT-PCR products covering the whole *Plasmodium yoelii* HECT E3 ligase gene. The arrows on top of the gels indicate primer positions and directions used in the amplification. YM and N67, amplified from cDNA samples of the two parental parasites; gDNA, amplification products from genomic YM DNA. Primers 15 and 16 produced an additional band smaller than that of the genomic band, suggesting alternative splicing.

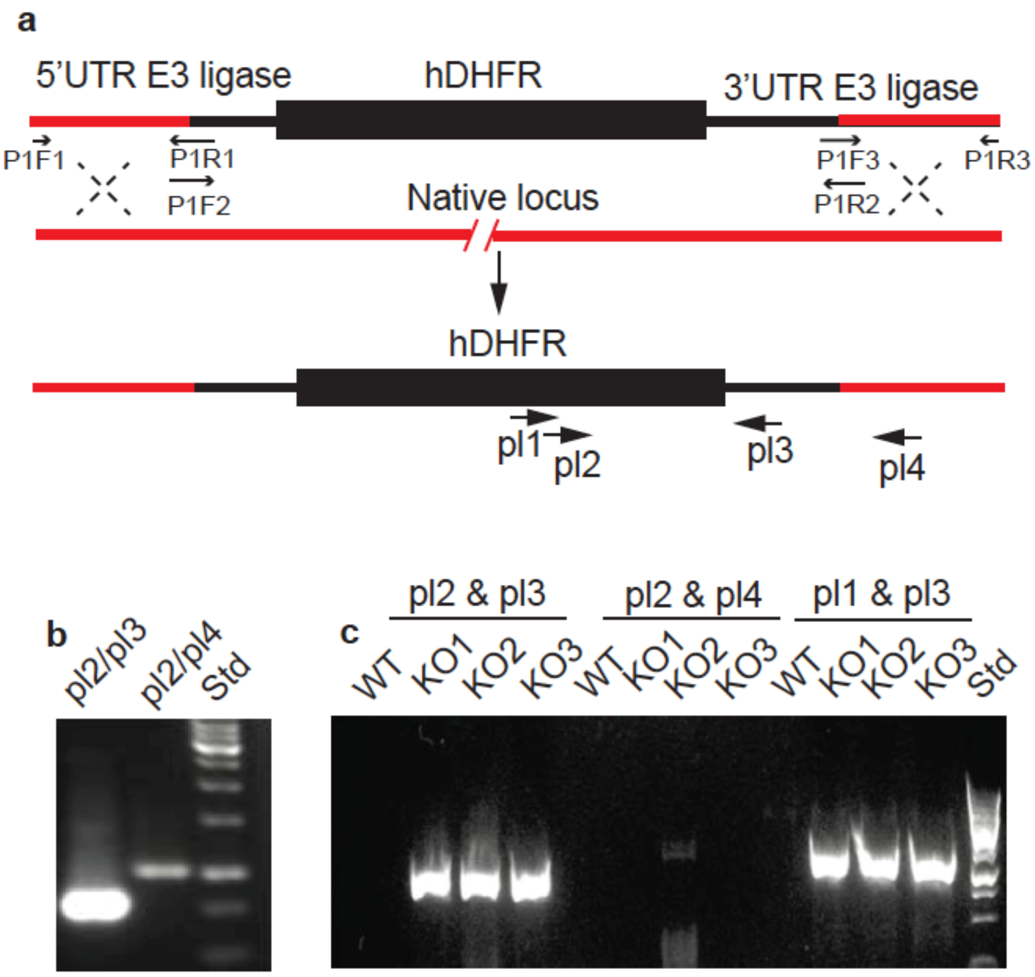

Supplementary Fig. 9. Attempts to disrupt the *Plasmodium yoelii* HECT E3 ubiquitin ligase gene. (**a**) Diagrams showing a linear construct for the insertion of a drug cassette into the HECT E3 ubiquitin ligase gene. The linear construct was made by assembly of PCR products using methods as described [3,4]. (**b**) PCR products from uncloned parasites before drug selection using primers as indicated in (**a**). (**c**) PCR products from parasites of three independent transfections after drug selection using primers as indicated.

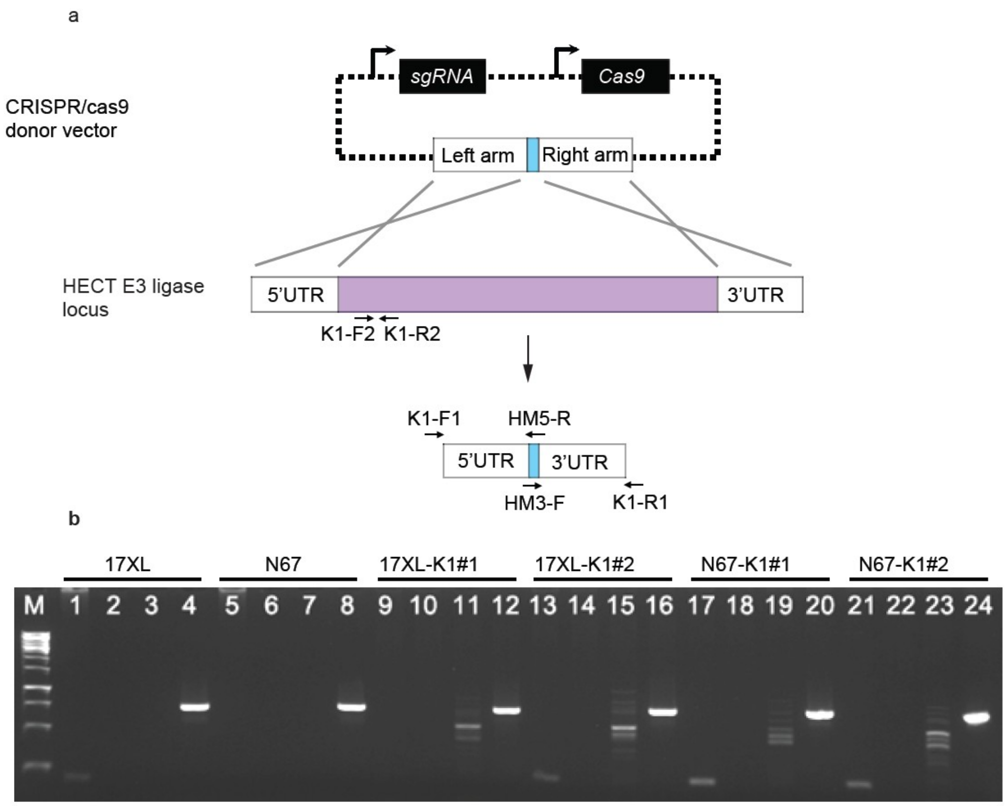

Supplementary Fig. 10. Attempts to disrupt the *Plasmodium yoelii* HECT E3 ubiquitin ligase gene. A CRISPR/Cas9 strategy was used to disrupt the gene as described [5]. (**a**) Diagram showing plasmid map with left and right arms from 5' untranslated region (5'UTR, 679 bp) and 3'UTR (699 bp) of the *Pyheul* gene as homologous DNA templates. Primers used in PCR amplification are as indicated, and their sequences are listed in Supplementary Table 5. The middle inserted sequence (HM5-R: CGATTAGCGTCCGCGGGGACCAT; HM3-F: ACGCTAATCGTAGCTAGCCTGCT) is used as a primer site for verification of sequence integration into parasite chromosome. (**b**) PCR products amplified from 17XL, N67, and uncloned transfected parasites. Primer pairs and gel lanes are as following: K1-F1/K1-R1 (lane 1, 5, 9, 13, 17, 21); K1-F1/HM5-R (lane 2, 6, 10, 14, 18, 22); HM3-F/K1-R1 (lane 3, 7, 11, 15, 19, 23); K1-F2/K1-R2 (lane 4, 8, 12, 16, 20, 24).

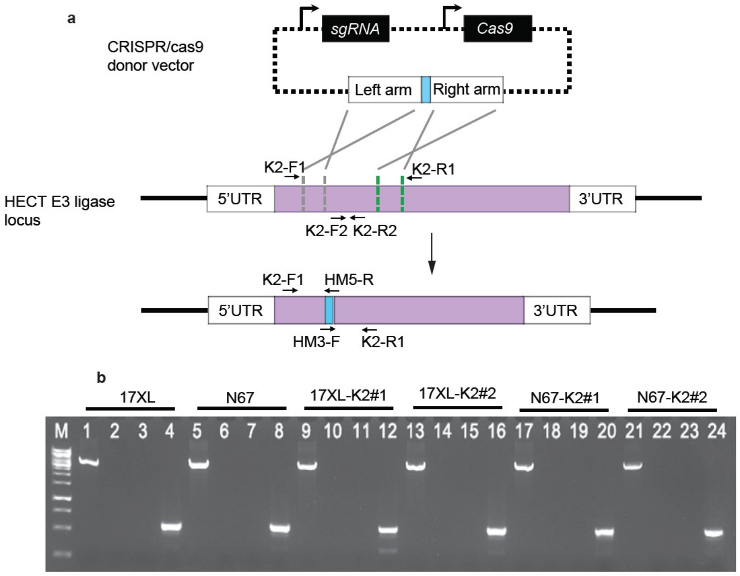

Supplementary Fig. 11. Attempts to replace partial *Plasmodium yoelii* HECT E3 ubiquitin ligase gene sequence to disrupt the gene. A CRISPR/Cas9 strategy was used to disrupt the *Pyheul* gene as described [5]. (**a**) Diagram showing plasmid map with left and right arms from the coding regions (left arm, 658 bp, position from 835-1,492 bp; right arm, 749 bp, from 3,866-4,614 bp) of the *Pyheul* gene as homologous DNA templates. Primers used in PCR amplification are as indicated, and their sequences are listed in Supplementary Table 5. The middle inserted sequence (HM5-R: CGATTAGCGTCCGCGGGACCAT; HM3-F: ACGCTAATCGTAGCTAGCCTGCT) is used as a primer site for verification of sequence integration into parasite chromosome. (**b**) PCR products amplified from 17XL, N67, and uncloned transfected parasites. Primer pair K2-F1/K2-R1, lane 1, 5, 9, 13, 17, 21; primer pair K2-F1/HM5-R, lane 2, 6, 10, 14, 18, 22; primer pair HM3-F/K2-R1, lane 3, 7, 11, 15, 19, 23; primer pair K2-F2/K2-R2, lane 4, 8, 12, 16, 20, 24.

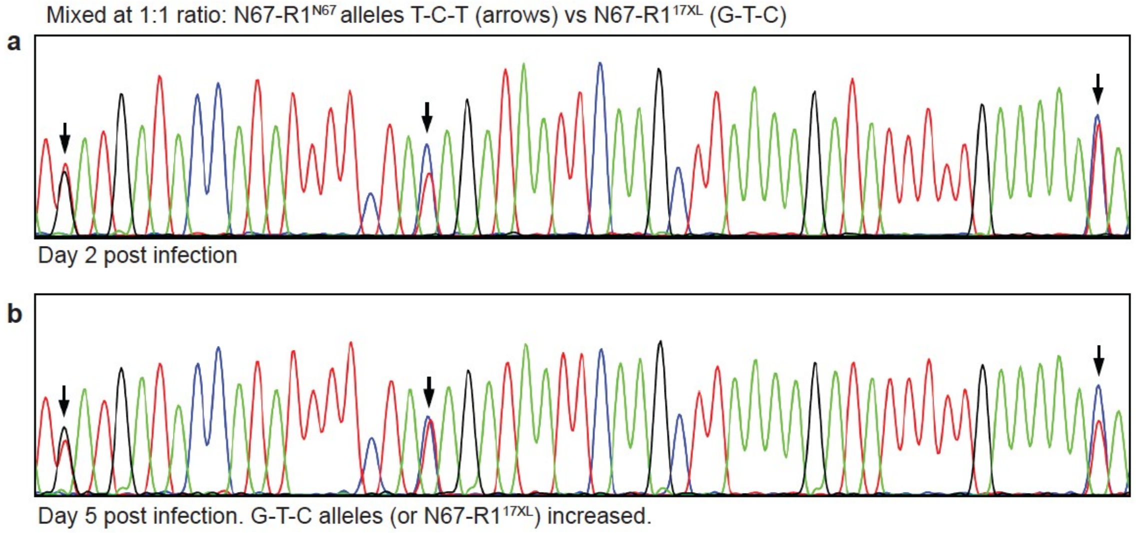

Supplementary Fig. 12. Electropherograms of DNA sequences from a mouse infected with a mixture of two isogenic parasites. Parasites (N67-R1^{17XL} and N67-R1^{N67}) at 1:1 ratio (1 × 10^5 each) were mixed and injected into a mouse. Bloods were collected on day 2 (**a**) and day 4 (**b**) post infection. DNA samples were prepared, PCR amplified the target region, and sequenced commercially. Similar results were obtained from a repeated experiment. Note the G-T-C alleles increased on day 4.

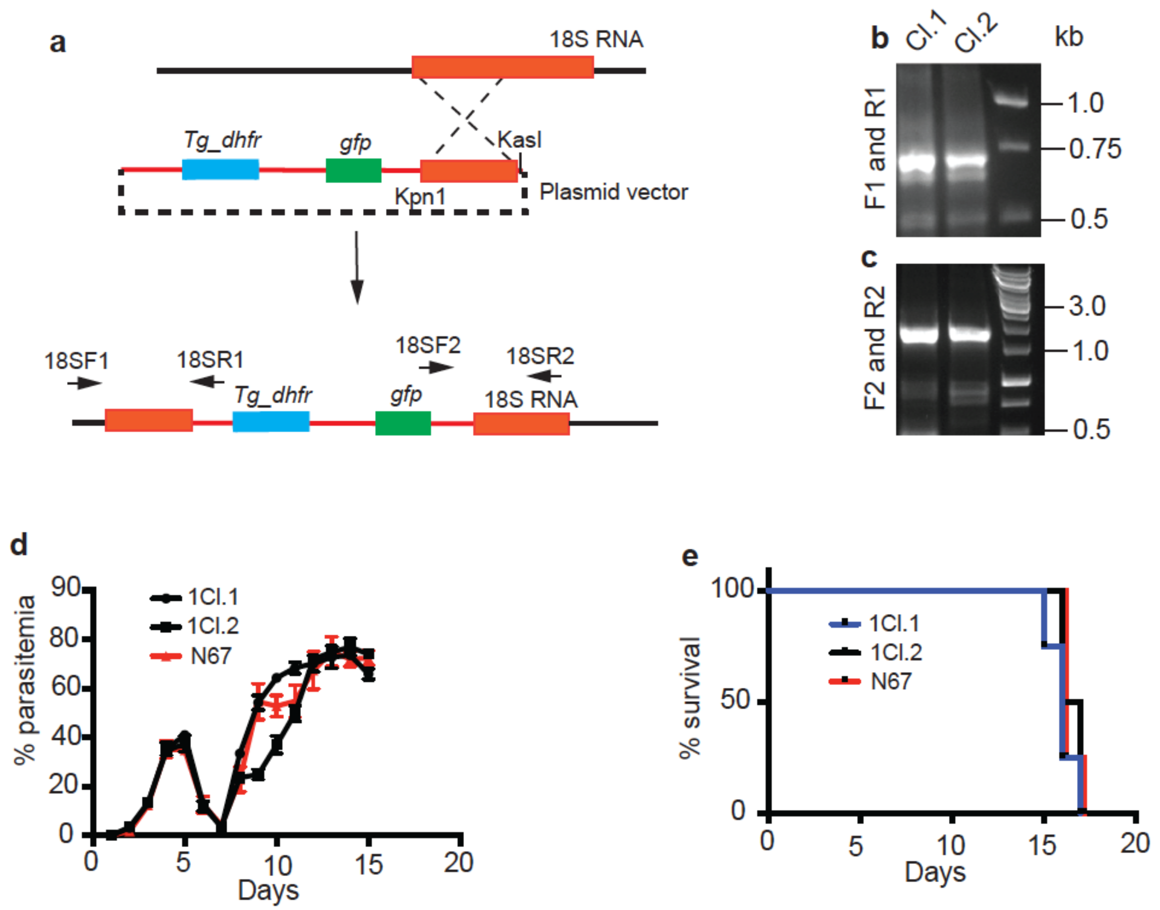

Supplementary Fig. 13. Insertion of a drug cassette into the 18S rRNA gene of *Plasmodium yoelii nigeriensis* N67. (**a**) A diagram showing the insertion sites of the drug cassette. *Tg_dhfr*, *Toxoplasma gondii* dihydrofolate reductase; *gfp*, green fluorescent protein. (**b-c**) PCR products from two parasite clones after drug selection and parasite cloning. Primers used are as indicated. In each case, one primer is on the parasite chromosome, and the other one is on the drug cassette. (**d**) Parasitemia of mice infected with N67 and the two parasite clones. (**e**), Mortality rate of N67 and the two clones.

Supplementary Table 1. Histological observa]ons of mouse organs infected with *Plasmodium y. nigeriensis* N67 and *P. yoelii yoelii* YM parasites

Pathology #	Animal #	Mouse strain	Parasite strain	Days post infection	Lung	Thymus	Liver	Spleen	Kidney	GI tract	Brain	Spinal cord
130184	3	C57BL/6	P. yoelii YM	4	there are increased numbers of mononuclear cells within the alveolar walls; capillaries are congested and contain pigmented RBCs and phagocytes;	n/a	there are multiple foci of acute hepatocelluar necrosis; there are moderate numbers of particle pigmented (hemozoin-laden) Kupffer cells, phagocytes and RBCs	there is mild/moderate lymphoid necrosis and increased numbers of particle pigmented (hemozoin-laden) macrophages and RBCs	wnl	wnl	few blood vessels are congested and contain increased numbers of pigmented RBCs	wnl
130185	4	C57BL/6	P. yoelii YM	4	there are increased numbers of mononuclear cells within the alveolar walls; capillaries are congested and contain pigmented RBCs and phagocytes; there is mild fibrin and edema in alveoli	There is moderate lymphoid necrosis	there are moderate numbers of particle pigmented (hemozoin-laden) Kupffer cells, phagocytes and RBCs	the spleen is enlarged and there is moderate lymphoid hyperplasia; increased numbers of particle pigmented (hemozoin-laden) macrophages and RBCs; and moderate EMH	there are large numbers of pigmented RBCs in vessels	wnl	there are a few small foci of hemorrhage	wnl
130186	3	C57BL/6	P. yoelii N67	4	there are increased numbers of mononuclear cells within the alveolar walls; capillaries are congested and contain pigmented RBCs and phagocytes;	wnl	there are moderate numbers of particle pigmented (hemozoin-laden) Kupffer cells, phagocytes and RBCs	the spleen is enlarged and there is mild/moderate lymphoid hyperplasia; increased numbers of particle pigmented (hemozoin-laden) macrophages and RBCs	wnl	wnl	wnl	wnl
130187	4	C57BL/6	P. yoelii N67	4	there are increased numbers of mononuclear cells within the alveolar walls; capillaries are congested and contain pigmented RBCs and phagocytes;	n/a	there are moderate numbers of particle pigmented (hemozoin-laden) Kupffer cells, phagocytes and RBCs; moderate EMH	the spleen is enlarged and there is mild/moderate lymphoid hyperplasia; increased numbers of particle pigmented (hemozoin-laden) macrophages and RBCs	there are large numbers of pigmented RBCs in vessels	wnl	wnl	wnl
130188	5	C57BL/6	P. yoelii YM	7	there are increased numbers of mononuclear cells within the alveolar walls; capillaries are congested and contain pigmented RBCs and phagocytes; there is mild edema and fibrin	There is mild lymphoid necrosis	there are moderate numbers of particle pigmented (hemozoin-laden) Kupffer cells, phagocytes and RBCs; moderate EMH	the spleen is enlarged and there is moderate lymphoid hyperplasia, histiocytosis and EMH	wnl	wnl	wnl	wnl
130189	6	C57BL/6	P. yoelii YM	7	there are increased numbers of mononuclear cells within the alveolar walls; capillaries are congested and contain pigmented RBCs and phagocytes;	There is mild lymphoid necrosis	there are moderate numbers of particle pigmented (hemozoin-laden) Kupffer cells, phagocytes and RBCs; extensive EMH	the spleen is very enlarged and extensive histiocytosis	wnl	wnl	there are a few small foci of hemorrhage	wnl
130190	7	C57BL/6	P. yoelii YM	7	wnl	There is severe lymphoid necrosis	there are multiple foci of acute hepatocelluar necrosis; there are moderate numbers of particle pigmented (hemozoin-laden) Kupffer cells, phagocytes and RBCs	the spleen is very enlarged and there is extensive histiocytosis with lymphoid hyperplasia	wnl	wnl	there is one small focus of hemorrhage	wnl
130191	5	C57BL/6	P. yoelii N67	7	multifocally, there is moderate to severe interstitial pneumonia	There is severe lymphoid necrosis	there are moderate numbers of particle pigmented (hemozoin-laden) Kupffer cells, phagocytes and RBCs; mild EMH	the spleen is very enlarged and there is extensive histiocytosis with lymphoid hyperplasia; increased numbers of particle pigmented (hemozoin-laden) macrophages	wnl	wnl	wnl	wnl
130192	6	C57BL/6	P. yoelii N67	7	there are increased numbers of mononuclear cells within the alveolar walls; capillaries are congested and contain pigmented RBCs and phagocytes;	There is severe lymphoid necrosis	there are moderate numbers of particle pigmented (hemozoin-laden) Kupffer cells, phagocytes and RBCs; mild EMH	the spleen is very enlarged and there is extensive histiocytosis with lymphoid hyperplasia; increased numbers of particle pigmented (hemozoin-laden) macrophages	wnl	wnl	there are a few small foci of hemorrhage	wnl
130193	7	C57BL/6	P. yoelii N67	7	there are increased numbers of mononuclear cells within the alveolar walls; capillaries are congested and contain pigmented RBCs and phagocytes;	There is severe lymphoid necrosis	there are moderate numbers of particle pigmented (hemozoin-laden) Kupffer cells, phagocytes and RBCs; mild EMH	the spleen is very enlarged and there is extensive histiocytosis with lymphoid hyperplasia; increased numbers of particle pigmented (hemozoin-laden) macrophages	wnl	w	wnl	wnl

EMH= extramedullary hematopoiesis
wnl, within normal limit

Supplementary Table 2. Microsatellite markers and their primer sequences used in the initial typing of the progeny

Marker	Sequence size (bp)	Py chr.	YM (bp)	N67 (bp)	Motif	Forward primer	Reverse primer
Py183	139	chr13	155	114	AT	5'TATCCTTTGTGTCAGGTAA3'	5'GTGAATAATTGAATAGGTTG3'
Py718	128	chr9	145	179	AAAT/AT	5'CACTTGATCTTTCCAACC3'	5'TGTTGAAATTGAAGAATCAC3'
Py1352	234	chr7	253	358	CAT	5'GACTTAAAATGTAGTTCAAG3'	5'ATGAATCCACTGACTTCG3'
Py1414	207	chr4	225	134	AAT	5'AACAAAACAGGTAGCAACA3'	5'ATCATGGTTTATATTCACAG3'
Py1458	138	chr14	156	104	AT	5'CGCTTTTTTATGCGTATAC3'	5'TTAAGTATCAATTGGACGG3'
Py1654	175	chr8	193	247	AT/T	5'TGACAACAATGGTAACTCT3'	5'TCGTTTCAATAAGGTCATC3'
Py1685	145	chr6	163	117	TATG/TA	5'AAAATTTAATCTTATCTCCGT3'	5'ATGTGTTACTCATGCGAC3'
Py1788	143	chr5	155	228	A	5'GATGCTAAAATAGAATTAGC3'	5'TGGTAATTTCGATTATCAGT3'
Py1836	172	chr10	189	123	AT	5'CTTACTATTCGCAAAGTTG3'	5'ATCTTCTTGTAGGTTTCGA3'
Py2260	238	chr12	243	201	CATA	5'TAGCAGCATATTCCTTGC3'	5'ATGGCTTCTTCCAATAAGT3'

Note: py chr, *Plasmodium yoelii* chromosome; YM (bp), PCR product sizes in basepairs from YM parasite; N67 (bp), PCR product sizes in base pair from N67 parasite; moAf, repeaAng moAfs of the microsatellites.

Supplementary Table 3. Candidate genes in the chromosome 1 locus

Gene ID	Genomic Location	Product Description	Protein Length	# TM Domains	SignalP Scores
PYYM_0115900	PyYM_01_v1: 622,336 - 624,444 (+)	YIR protein	632	2	No
PYYM_0116000	PyYM_01_v1: 624,944 - 626,063 (-)	fam-a protein	205	0	No
PYYM_0116100	PyYM_01_v1: 628,961 - 629,639 (-)	fam-a protein	146	0	Yes
PYYM_0116200	PyYM_01_v1: 632,522 - 636,127 (+)	YIR protein	1131	2	No
PYYM_0116300	PyYM_01_v1: 636,951 - 638,074 (-)	fam-a protein	224	0	Yes

Supplementary Table 3. Candidate genes in the chromosome 8 locus

PYYM_0800300	PyYM_08_v1: 10,030 - 11,045 (-)	YIR protein	270	1	No
PYYM_0800400	PyYM_08_v1: 13,672 - 14,969 (-)	YIR protein	264	0	No
PYYM_0800500	PyYM_08_v1: 17,880 - 18,984 (-)	YIR protein	303	1	No
PYYM_0800600	PyYM_08_v1: 21,744 - 22,913 (-)	YIR protein	309	1	No
PYYM_0800700	PyYM_08_v1: 25,194 - 26,209 (-)	YIR protein	269	1	No
PYYM_0800800	PyYM_08_v1: 28,764 - 29,951 (-)	YIR protein	320	1	No
PYYM_0800900	PyYM_08_v1: 32,345 - 33,491 (-)	YIR protein	307	1	No

SupplementaryTable 4. Nucleotide and amino acid differences in the *Plasmodium yoelii* HECT E3 ligase gene between *Plamodium y. nigeriensis* N67 and *P. y. yoelii* YM.

Chromsome 7	Position	Cl.13	Cl.35	Cl.49	YM	N67	Depth	Coding-synonymous_nonsynonymous	Mutation	Codon change	AA change
PyYM_07_v1	182821	182821	182821	182821	A	G	DP=330	SYNONYMOUS_CODING	SILENT	tcA/tcG	S52
PyYM_07_v1	182940	182940	182940	182940	G	A	DP=219	NON_SYNONYMOUS_CODING	MISSENSE	gGc/gAc	G92D
PyYM_07_v1	182941	182941	182941	182941	C	T	DP=216	SYNONYMOUS_CODING	SILENT	ggC/ggT	G92
PyYM_07_v1	182944	182944	182944	182944	T	C	DP=236	SYNONYMOUS_CODING	SILENT	aaT/aaC	N93
PyYM_07_v1	182960	182960	182960	182960	A	G	DP=281	NON_SYNONYMOUS_CODING	MISSENSE	Aca/Gca	T99A
PyYM_07_v1	182963	182963	182963	182963	T	A	DP=275	NON_SYNONYMOUS_CODING	MISSENSE	Tgt/Agt	C100S
PyYM_07_v1	182965	182965	182965	182965	T	C	DP=278	SYNONYMOUS_CODING	SILENT	tgT/tgC	C100
PyYM_07_v1	182974	182974	182974	182974	C	T	DP=319	SYNONYMOUS_CODING	SILENT	atC/atT	I103
PyYM_07_v1	182995	182995	182995	182995	G	A	DP=356	SYNONYMOUS_CODING	SILENT	caG/caA	Q110
PyYM_07_v1	183049	183049	183049	183049	T	C	DP=357	SYNONYMOUS_CODING	SILENT	aaT/aaC	N128
PyYM_07_v1	183166	183166	183166	183166	G	C	DP=325	SYNONYMOUS_CODING	SILENT	acG/acC	T167
PyYM_07_v1	183293	183293	183293	183293	A	G	DP=336	NON_SYNONYMOUS_CODING	MISSENSE	Ata/Gta	I210V
PyYM_07_v1	183580	183580	183580	183580	A	G	DP=336	SYNONYMOUS_CODING	SILENT	aaA/aaG	K305
PyYM_07_v1	183607	183607	183607	183607	T	C	DP=345	SYNONYMOUS_CODING	SILENT	tgT/tgC	C314
PyYM_07_v1	184015	184015	184015	184015	C	A	DP=369	NON_SYNONYMOUS_CODING	MISSENSE	ttC/ttA	F450L
PyYM_07_v1	184024	184024	184024	184024	T	C	DP=366	SYNONYMOUS_CODING	SILENT	tcT/tcC	S453
PyYM_07_v1	184026	184026	184026	184026	T	C	DP=365	NON_SYNONYMOUS_CODING	MISSENSE	aTa/aCa	I454T
PyYM_07_v1	184091	184091	184091	184091	G	A	DP=347	NON_SYNONYMOUS_CODING	MISSENSE	Gtt/Att	V476I
PyYM_07_v1	184161	184161	184161	184161	A	T	DP=336	NON_SYNONYMOUS_CODING	MISSENSE	cAt/cTt	H499L
PyYM_07_v1	184173	184173	184173	184173	G	C	DP=349	NON_SYNONYMOUS_CODING	MISSENSE	gGt/gCt	G503A
PyYM_07_v1	184186	184186	184186	184186	G	A	DP=346	SYNONYMOUS_CODING	SILENT	tcG/tcA	S507
PyYM_07_v1	184261	184261	184261	184261	C	T	DP=309	SYNONYMOUS_CODING	SILENT	gaC/gaT	D532
PyYM_07_v1	184286	184286	184286	184286	G	A	DP=269	NON_SYNONYMOUS_CODING	MISSENSE	Gta/Ata	V541I
PyYM_07_v1	184295	184295	184295	184295	T	C	DP=264	NON_SYNONYMOUS_CODING	MISSENSE	Tca/Cca	S544P
PyYM_07_v1	184314	184314	184314	184314	T	C	DP=283	NON_SYNONYMOUS_CODING	MISSENSE	tTa/tCa	L550S
PyYM_07_v1	184318	184318	184318	184318	T	C	DP=292	SYNONYMOUS_CODING	SILENT	caT/caC	H551
PyYM_07_v1	184340	184340	184340	184340	G	T	DP=301	NON_SYNONYMOUS_CODING	MISSENSE	Gta/Tta	V559L
PyYM_07_v1	184341	184341	184341	184341	T	C	DP=305	NON_SYNONYMOUS_CODING	MISSENSE	gTa/gCa	V559A
PyYM_07_v1	184772	184772	184772	184772	C	T	DP=294	SYNONYMOUS_CODING	SILENT	Cta/Tta	L703
PyYM_07_v1	184861	184861	184861	184861	A	G	DP=381	SYNONYMOUS_CODING	SILENT	gaA/gaG	E732
PyYM_07_v1	184987	184987	184987	184987	C	T	DP=323	SYNONYMOUS_CODING	SILENT	gaC/gaT	D774
PyYM_07_v1	185061	185061	185061	185061	GTAATAA	GTAATA	DP=229	CODON_CHANGE_PLUS_CODON_DELETION		aataatgataatacc/acc	NNDNT801 T
PyYM_07_v1	185101	185101	185101	185101	C	T	DP=285	SYNONYMOUS_CODING	SILENT	agC/agT	S812
PyYM_07_v1	185117	185117	185117	185117	A	T	DP=280	NON_SYNONYMOUS_CODING	MISSENSE	Acc/Tcc	T818S
PyYM_07_v1	185189	185189	185189	185189	G	T	DP=332	NON_SYNONYMOUS_CODING	MISSENSE	Gat/Tat	D842Y
PyYM_07_v1	185208	185208	185208	185208	T	C	DP=370	NON_SYNONYMOUS_CODING	MISSENSE	aTa/aCa	I848T
PyYM_07_v1	185243	185243	185243	185243	C	T	DP=386	NON_SYNONYMOUS_CODING	MISSENSE	Cac/Tac	H860Y
PyYM_07_v1	185395	185395	185395	185395	A	T	DP=314	SYNONYMOUS_CODING	SILENT	acA/acT	T910
PyYM_07_v1	185431	185431	185431	185431	A	C	DP=295	SYNONYMOUS_CODING	SILENT	gtA/gtC	V922
PyYM_07_v1	185467	185467	185467	185467	A	G	DP=310	SYNONYMOUS_CODING	SILENT	aaA/aaG	K934
PyYM_07_v1	185623	185623	185623	185623	T	A	DP=340	SYNONYMOUS_CODING	SILENT	atT/atA	I986
PyYM_07_v1	185695	185695	185695	185695	T	C	DP=321	SYNONYMOUS_CODING	SILENT	taT/taC	Y1010
PyYM_07_v1	185794	185794	185794	185794	C	T	DP=369	SYNONYMOUS_CODING	SILENT	aaC/aaT	N1043

PyYM_07_v1	186412	186412	186412	186412	A	C	DP=329	NON_SYNONYMOUS_CODING	MISSENSE	ttA/ttC	L1249F
PyYM_07_v1	186563	186563	186563	186563	T	C	DP=321	SYNONYMOUS_CODING	SILENT	Tta/Cta	L1300
PyYM_07_v1	187006	187006	187006	187006	C	T	DP=324	SYNONYMOUS_CODING	SILENT	gtC/gtT	V1447
PyYM_07_v1	187102	187102	187102	187102	T	C	DP=342	SYNONYMOUS_CODING	SILENT	taT/taC	Y1479
PyYM_07_v1	187330	187330	187330	187330	C	T	DP=305	SYNONYMOUS_CODING	SILENT	aaC/aaT	N1555
PyYM_07_v1	187681	187681	187681	187681	C	A	DP=321	SYNONYMOUS_CODING	SILENT	tcC/tcA	S1672
PyYM_07_v1	187832	187832	187832	187832	G	C	DP=309	NON_SYNONYMOUS_CODING	MISSENSE	Gaa/Caa	E1723Q
PyYM_07_v1	188005	188005	188005	188005	G	A	DP=334	SYNONYMOUS_CODING	SILENT	gtG/gtA	V1780
PyYM_07_v1	188061	188061	188061	188061	C	T	DP=274	NON_SYNONYMOUS_CODING	MISSENSE	cCc/cTc	P1799L
PyYM_07_v1	188123	188123	188123	188123	A	C	DP=315	NON_SYNONYMOUS_CODING	MISSENSE	Aag/Cag	K1820Q
PyYM_07_v1	188147	188147	188147	188147	C	A	DP=312	NON_SYNONYMOUS_CODING	MISSENSE	Cac/Aac	H1828N
PyYM_07_v1	188176	188176	188176	188176	T	C	DP=306	SYNONYMOUS_CODING	SILENT	aaT/aaC	N1837
PyYM_07_v1	188182	188182	188182	188182	A	G	DP=289	NON_SYNONYMOUS_CODING	MISSENSE	atA/atG	I1839M
PyYM_07_v1	188284	188284	188284	188284	T	C	DP=358	SYNONYMOUS_CODING	SILENT	ccT/ccC	P1873
PyYM_07_v1	188423	188423	188423	188423	C	T	DP=254	NON_SYNONYMOUS_CODING	MISSENSE	Cct/Tct	P1920S
PyYM_07_v1	188544	188544	188544	188544	A	G	DP=299	NON_SYNONYMOUS_CODING	MISSENSE	aAt/aGt	N1960S
PyYM_07_v1	188596	188596	188596	188596	A	G	DP=369	SYNONYMOUS_CODING	SILENT	ttA/ttG	L1977
PyYM_07_v1	188767	188767	188767	188767	C	T	DP=247	SYNONYMOUS_CODING	SILENT	aaC/aaT	N2034
PyYM_07_v1	189067	189067	189067	189067	C	T	DP=313	SYNONYMOUS_CODING	SILENT	aaC/aaT	N2134
PyYM_07_v1	189073	189073	189073	189073	A	T	DP=302	NON_SYNONYMOUS_CODING	MISSENSE	caA/caT	Q2136H
PyYM_07_v1	189091	189091	189091	189091	T	C	DP=293	SYNONYMOUS_CODING	SILENT	aaT/aaC	N2142
PyYM_07_v1	189106	189106	189106	189106	C	T	DP=308	SYNONYMOUS_CODING	SILENT	gaC/gaT	D2147
PyYM_07_v1	189117	189117	189117	189117	T	C	DP=275	NON_SYNONYMOUS_CODING	MISSENSE	gTc/gCc	V2151A
PyYM_07_v1	189118	189118	189118	189118	C	T	DP=276	SYNONYMOUS_CODING	SILENT	gtC/gtT	V2151
PyYM_07_v1	189120	189120	189120	189120	G	A	DP=280	NON_SYNONYMOUS_CODING	MISSENSE	aGt/aAt	S2152N
PyYM_07_v1	189125	189125	189125	189125	G	A	DP=319	NON_SYNONYMOUS_CODING	MISSENSE	Gtt/Att	V2154I
PyYM_07_v1	189128	189128	189128	189128	T	C	DP=317	NON_SYNONYMOUS_CODING	MISSENSE	Tca/Cca	S2155P
PyYM_07_v1	189154	189154	189154	189154	C	T	DP=300	SYNONYMOUS_CODING	SILENT	gaC/gaT	D2163
PyYM_07_v1	189253	189253	189253	189253	G	C	DP=383	SYNONYMOUS_CODING	SILENT	tcG/tcC	S2196
PyYM_07_v1	189436	189436	189436	189436	T	C	DP=309	SYNONYMOUS_CODING	SILENT	ttT/ttC	F2257
PyYM_07_v1	189930	189930	189930	189930	C	T	DP=374	NON_SYNONYMOUS_CODING	MISSENSE	cCa/cTa	P2422L
PyYM_07_v1	190087	190087	190087	190087	C	T	DP=377	SYNONYMOUS_CODING	SILENT	aaC/aaT	N2474
PyYM_07_v1	190158	190158	190158	190158	T	C	DP=323	NON_SYNONYMOUS_CODING	MISSENSE	aTa/aCa	I2498T
PyYM_07_v1	190432	190432	190432	190432	A	G	DP=305	SYNONYMOUS_CODING	SILENT	aaA/aaG	K2589
PyYM_07_v1	190843	190843	190843	190843	A	C	DP=170	NON_SYNONYMOUS_CODING	MISSENSE	caA/caC	Q2726H
PyYM_07_v1	190856	190856	190856	190856	G	A	DP=204	NON_SYNONYMOUS_CODING	MISSENSE	Gca/Aca	A2731T
PyYM_07_v1	190882	190882	190882	190882	T	C	DP=320	SYNONYMOUS_CODING	SILENT	atT/atC	I2739
PyYM_07_v1	190904	190904	190904	190904	G	A	DP=312	NON_SYNONYMOUS_CODING	MISSENSE	Ggt/Agt	G2747S
PyYM_07_v1	190905	190905	190905	190905	G	A	DP=308	NON_SYNONYMOUS_CODING	MISSENSE	gGt/gAt	G2747D
PyYM_07_v1	190982	190982	190982	190982	G	C	DP=281	NON_SYNONYMOUS_CODING	MISSENSE	Gca/Cca	A2773P
PyYM_07_v1	191026	191026	191026	191026	A	G	DP=294	SYNONYMOUS_CODING	SILENT	tcA/tcG	S2787
PyYM_07_v1	191083	191083	191083	191083	C	G	DP=308	NON_SYNONYMOUS_CODING	MISSENSE	gaC/gaG	D2806E
PyYM_07_v1	191107	191107	191107	191107	T	C	DP=334	SYNONYMOUS_CODING	SILENT	atT/atC	I2814
PyYM_07_v1	191115	191115	191115	191115	G	A	DP=345	NON_SYNONYMOUS_CODING	MISSENSE	aGt/aAt	S2817N
PyYM_07_v1	191125	191125	191125	191125	T	C	DP=345	SYNONYMOUS_CODING	SILENT	ccT/ccC	P2820
PyYM_07_v1	191179	191179	191179	191179	T	C	DP=388	SYNONYMOUS_CODING	SILENT	aaT/aaC	N2838
PyYM_07_v1	191205	191205	191205	191205	G	T	DP=358	NON_SYNONYMOUS_CODING	MISSENSE	gGt/gTt	G2847V
PyYM_07_v1	191285	191285	191285	191285	C	A	DP=296	NON_SYNONYMOUS_CODING	MISSENSE	Cta/Ata	L2874I
PyYM_07_v1	191461	191461	191461	191461	T	C	DP=344	SYNONYMOUS_CODING	SILENT	taT/taC	Y2932
PyYM_07_v1	191658	191658	191658	191658	T	C	DP=353	NON_SYNONYMOUS_CODING	MISSENSE	gTt/gCt	V2998A
PyYM_07_v1	191662	191662	191662	191662	C	T	DP=348	SYNONYMOUS_CODING	SILENT	aaC/aaT	N2999

PyYM_07_v1	191758	191758	191758	191758	G	A	DP=263	SYNONYMOUS_CODING	SILENT	agG/agA	R3031
PyYM_07_v1	191791	191791	191791	191791	T	A	DP=313	SYNONYMOUS_CODING	SILENT	ccT/ccA	P3042
PyYM_07_v1	191803	191803	191803	191803	G	T	DP=313	SYNONYMOUS_CODING	SILENT	acG/acT	T3046
PyYM_07_v1	191809	191809	191809	191809	T	G	DP=302	SYNONYMOUS_CODING	SILENT	tcT/tcG	S3048
PyYM_07_v1	191828	191828	191828	191828	A	G	DP=318	NON_SYNONYMOUS_CODING	MISSENSE	Att/Gtt	I3055V
PyYM_07_v1	191836	191836	191836	191836	C	T	DP=309	SYNONYMOUS_CODING	SILENT	aaC/aaT	N3057
PyYM_07_v1	191841	191841	191841	191841	T	C	DP=316	NON_SYNONYMOUS_CODING	MISSENSE	aTt/aCt	I3059T
PyYM_07_v1	191850	191850	191850	191850	C	T	DP=331	NON_SYNONYMOUS_CODING	MISSENSE	gCt/gTt	A3062V
PyYM_07_v1	191861	191861	191861	191861	A	G	DP=330	NON_SYNONYMOUS_CODING	MISSENSE	Att/Gtt	I3066V
PyYM_07_v1	191938	191938	191938	191938	C	T	DP=343	SYNONYMOUS_CODING	SILENT	ggC/ggT	G3091
PyYM_07_v1	191945	191945	191945	191945	G	A	DP=336	NON_SYNONYMOUS_CODING	MISSENSE	Gtt/Att	V3094I
PyYM_07_v1	191982	191982	191982	191982	G	A	DP=330	NON_SYNONYMOUS_CODING	MISSENSE	aGt/aAt	S3106N
PyYM_07_v1	192334	192334	192334	192334	A	G	DP=235	SYNONYMOUS_CODING	SILENT	gaA/gaG	E3223
PyYM_07_v1	192553	192553	192553	192553	C	T	DP=325	SYNONYMOUS_CODING	SILENT	gcC/gcT	A3296
PyYM_07_v1	192655	192655	192655	192655	C	T	DP=344	SYNONYMOUS_CODING	SILENT	ttC/ttT	F3330
PyYM_07_v1	192663	192663	192663	192663	G	A	DP=352	NON_SYNONYMOUS_CODING	MISSENSE	tGc/tAc	C3333Y
PyYM_07_v1	192805	192805	192805	192805	A	G	DP=332	SYNONYMOUS_CODING	SILENT	aaA/aaG	K3380
PyYM_07_v1	192974	192974	192974	192974	G	A	DP=352	NON_SYNONYMOUS_CODING	MISSENSE	Gct/Act	A3437T
PyYM_07_v1	192993	192993	192993	192993	G	A	DP=352	NON_SYNONYMOUS_CODING	MISSENSE	aGc/aAc	S3443N
PyYM_07_v1	193006	193006	193006	193006	A	G	DP=361	SYNONYMOUS_CODING	SILENT	tcA/tcG	S3447
PyYM_07_v1	193132	193132	193132	193132	G	A	DP=383	SYNONYMOUS_CODING	SILENT	caG/caA	Q3489
PyYM_07_v1	193245	193245	193245	193245	C	T	DP=340	NON_SYNONYMOUS_CODING	MISSENSE	tCa/tTa	S3527L
PyYM_07_v1	193246	193246	193246	193246	A	G	DP=338	SYNONYMOUS_CODING	SILENT	tcA/tcG	S3527
PyYM_07_v1	193453	193453	193453	193453	C	T	DP=356	SYNONYMOUS_CODING	SILENT	gaC/gaT	D3596
PyYM_07_v1	193478	193478	193478	193478	C	G	DP=351	NON_SYNONYMOUS_CODING	MISSENSE	Ctt/Gtt	L3605V
PyYM_07_v1	193489	193489	193489	193489	A	G	DP=350	SYNONYMOUS_CODING	SILENT	ggA/ggG	G3608
PyYM_07_v1	193569	193569	193569	193569	G	A	DP=344	NON_SYNONYMOUS_CODING	MISSENSE	gGt/gAt	G3635D
PyYM_07_v1	193615	193615	193615	193615	G	A	DP=340	SYNONYMOUS_CODING	SILENT	ttG/ttA	L3650
PyYM_07_v1	193633	193633	193633	193633	A	C	DP=340	SYNONYMOUS_CODING	SILENT	atA/atC	I3656
PyYM_07_v1	193750	193750	193750	193750	T	C	DP=327	SYNONYMOUS_CODING	SILENT	aaT/aaC	N3695
PyYM_07_v1	193778	193778	193778	193778	C	A	DP=332	SYNONYMOUS_CODING	SILENT	Cga/Aga	R3705
PyYM_07_v1	193873	193873	193873	193873	G	A	DP=311	SYNONYMOUS_CODING	SILENT	ttG/ttA	L3736
PyYM_07_v1	193969	193969	193969	193969	T	C	DP=341	SYNONYMOUS_CODING	SILENT	taT/taC	Y3768
PyYM_07_v1	194029	194029	194029	194029	T	C	DP=403	SYNONYMOUS_CODING	SILENT	aaT/aaC	N3788
PyYM_07_v1	194107	194107	194107	194107	C	A	DP=344	SYNONYMOUS_CODING	SILENT	ctC/ctA	L3814
PyYM_07_v1	194172	194172	194172	194172	C	T	DP=270	NON_SYNONYMOUS_CODING	MISSENSE	aCt/aTt	T3836I
PyYM_07_v1	194458	194458	194458	194458	C	T	DP=294	SYNONYMOUS_CODING	SILENT	tgC/tgT	C3931
PyYM_07_v1	195027	195027	195027	195027	C	T	DP=339	NON_SYNONYMOUS_CODING	MISSENSE	aCa/aTa	T4121I
PyYM_07_v1	195178	195178	195178	195178	T	C	DP=324	SYNONYMOUS_CODING	SILENT	gtT/gtC	V4171
PyYM_07_v1	195201	195201	195201	195201	A	G	DP=324	NON_SYNONYMOUS_CODING	MISSENSE	aAt/aGt	N4179S
PyYM_07_v1	195415	195415	195415	195415	C	A	DP=280	SYNONYMOUS_CODING	SILENT	ccC/ccA	P4250
PyYM_07_v1	195424	195424	195424	195424	T	C	DP=259	SYNONYMOUS_CODING	SILENT	aaT/aaC	N4253
PyYM_07_v1	195745	195745	195745	195745	T	C	DP=308	SYNONYMOUS_CODING	SILENT	gaT/gaC	D4360
PyYM_07_v1	195859	195859	195859	195859	C	T	DP=504	SYNONYMOUS_CODING	SILENT	gaC/gaT	D4398
PyYM_07_v1	196000	196000	196000	196000	C	A	DP=386	NON_SYNONYMOUS_CODING	MISSENSE	gaC/gaA	D4445E
PyYM_07_v1	196129	196129	196129	196129	G	A	DP=244	SYNONYMOUS_CODING	SILENT	gaG/gaA	E4488
PyYM_07_v1	196153	196153	196153	196153	CGATGAT	CGATGATGA	DP=233	CODON_CHANGE_PLUS_CODON_DELETION		gatgaa/gaa	DE4502E
PyYM_07_v1	196399	196399	196399	196399	G	T	DP=436	SYNONYMOUS_CODING	SILENT	tcG/tcT	S4578
PyYM_07_v1	196431	196431	196431	196431	G	A	DP=369	NON_SYNONYMOUS_CODING	MISSENSE	aGt/aAt	S4589N
PyYM_07_v1	196744	196744	196744	196744	C	T	DP=407	SYNONYMOUS_CODING	SILENT	aaC/aaT	N4693
PyYM_07_v1	196858	196858	196858	196858	G	A	DP=411	SYNONYMOUS_CODING	SILENT	aaG/aaA	K4731

PyYM_07_v1	196910	196910	196910	196910	G	A	DP=349	NON_SYNONYMOUS_CODING	MISSENSE	Gac/Aac	D4749N
PyYM_07_v1	196918	196918	196918	196918	T	G	DP=329	NON_SYNONYMOUS_CODING	MISSENSE	aaT/aaG	N4751K
PyYM_07_v1	196995	196995	196995	196995	A	G	DP=343	NON_SYNONYMOUS_CODING	MISSENSE	cAt/cGt	H4777R
PyYM_07_v1	197170	197170	197170	197170	T	C	DP=368	SYNONYMOUS_CODING	SILENT	tcT/tcC	S4835
PyYM_07_v1	197297	197297	197297	197297	A	C	DP=264	NON_SYNONYMOUS_CODING	MISSENSE	Ata/Cta	I4878L
PyYM_07_v1	197336	197336	197336	197336	A	G	DP=286	NON_SYNONYMOUS_CODING	MISSENSE	Aca/Gca	T4891A
PyYM_07_v1	197391	197391	197391	197391	T	C	DP=314	NON_SYNONYMOUS_CODING	MISSENSE	aTa/aCa	I4909T
PyYM_07_v1	197407	197407	197407	197407	C	T	DP=313	SYNONYMOUS_CODING	SILENT	gaC/gaT	D4914
PyYM_07_v1	197500	197500	197500	197500	C	T	DP=357	SYNONYMOUS_CODING	SILENT	gaC/gaT	D4945
PyYM_07_v1	197624	197624	197624	197624	G	A	DP=354	NON_SYNONYMOUS_CODING	MISSENSE	Gtt/Att	V4987I
PyYM_07_v1	197704	197704	197704	197704	A	G	DP=275	SYNONYMOUS_CODING	SILENT	ccA/ccG	P5013
PyYM_07_v1	198022	198022	198022	198022	C	T	DP=355	SYNONYMOUS_CODING	SILENT	aaC/aaT	N5119
PyYM_07_v1	198167	198167	198167	198167	A	T	DP=391	NON_SYNONYMOUS_CODING	MISSENSE	Ata/Tta	I5168L
PyYM_07_v1	198200	198200	198200	198200	G	A	DP=394	NON_SYNONYMOUS_CODING	MISSENSE	Gtt/Att	V5179I
PyYM_07_v1	198245	198245	198245	198245	A	G	DP=376	NON_SYNONYMOUS_CODING	MISSENSE	Atg/Gtg	M5194V
PyYM_07_v1	198373	198373	198373	198373	T	C	DP=378	SYNONYMOUS_CODING	SILENT	gtT/gtC	V5236
PyYM_07_v1	198511	198511	198511	198511	T	C	DP=355	SYNONYMOUS_CODING	SILENT	gaT/gaC	D5282
PyYM_07_v1	198811	198811	198811	198811	T	C	DP=352	SYNONYMOUS_CODING	SILENT	gcT/gcC	A5382
PyYM_07_v1	198849	198849	198849	198849	T	C	DP=285	NON_SYNONYMOUS_CODING	MISSENSE	cTt/cCt	L5395P
PyYM_07_v1	198911	198911	198911	198911	A	G	DP=323	NON_SYNONYMOUS_CODING	MISSENSE	Atc/Gtc	I5416V
PyYM_07_v1	198953	198953	198953	198953	A	T	DP=346	NON_SYNONYMOUS_CODING	MISSENSE	Ata/Tta	I5430L
PyYM_07_v1	198958	198958	198958	198958	T	C	DP=353	SYNONYMOUS_CODING	SILENT	aaT/aaC	N5431
PyYM_07_v1	198960	198960	198960	198960	G	A	DP=354	NON_SYNONYMOUS_CODING	MISSENSE	gGc/gAc	G5432D
PyYM_07_v1	199102	199102	199102	199102	C	T	DP=318	SYNONYMOUS_CODING	SILENT	aaC/aaT	N5479
PyYM_07_v1	199111	199111	199111	199111	C	T	DP=323	SYNONYMOUS_CODING	SILENT	gaC/gaT	D5482
PyYM_07_v1	199537	199537	199537	199537	C	A	DP=288	SYNONYMOUS_CODING	SILENT	acC/acA	T5624
PyYM_07_v1	199601	199601	199601	199601	A	C	DP=324	NON_SYNONYMOUS_CODING	MISSENSE	Aat/Cat	N5646H
PyYM_07_v1	199708	199708	199708	199708	C	T	DP=385	SYNONYMOUS_CODING	SILENT	ggC/ggT	G5681
PyYM_07_v1	199713	199713	199713	199713	G	A	DP=398	NON_SYNONYMOUS_CODING	MISSENSE	aGa/aAa	R5683K
PyYM_07_v1	199907	199907	199907	199907	C	T	DP=361	NON_SYNONYMOUS_CODING	MISSENSE	Cca/Tca	P5748S
PyYM_07_v1	199976	199976	199976	199976	G	A	DP=372	NON_SYNONYMOUS_CODING	MISSENSE	Gca/Aca	A5771T
PyYM_07_v1	200010	200010	200010	200010	A	G	DP=374	NON_SYNONYMOUS_CODING	MISSENSE	aAt/aGt	N5782S
PyYM_07_v1	200014	200014	200014	200014	C	T	DP=374	SYNONYMOUS_CODING	SILENT	ggC/ggT	G5783
PyYM_07_v1	200016	200016	200016	200016	C	A	DP=375	NON_SYNONYMOUS_CODING	MISSENSE	cCa/cAa	P5784Q
PyYM_07_v1	200034	200034	200034	200034	A	T	DP=349	NON_SYNONYMOUS_CODING	MISSENSE	gAa/gTa	E5790V
PyYM_07_v1	200041	200041	200041	200041	A	C	DP=152	NON_SYNONYMOUS_CODING	MISSENSE	agA/agC	R5792S
PyYM_07_v1	200158	200158	200158	200158	T	C	DP=384	SYNONYMOUS_CODING	SILENT	gaT/gaC	D5831
PyYM_07_v1	200269	200269	200269	200269	G	A	DP=339	SYNONYMOUS_CODING	SILENT	ggG/ggA	G5868
PyYM_07_v1	200338	200338	200338	200338	C	T	DP=336	SYNONYMOUS_CODING	SILENT	atC/atT	I5891
PyYM_07_v1	200359	200359	200359	200359	A	G	DP=370	SYNONYMOUS_CODING	SILENT	tcA/tcG	S5898
PyYM_07_v1	200380	200380	200380	200380	A	G	DP=377	SYNONYMOUS_CODING	SILENT	tcA/tcG	S5905
PyYM_07_v1	200425	200425	200425	200425	A	G	DP=391	SYNONYMOUS_CODING	SILENT	ggA/ggG	G5920
PyYM_07_v1	200832	200832	200832	200832	G	A	DP=327	NON_SYNONYMOUS_CODING	MISSENSE	aGc/aAc	S6056N
PyYM_07_v1	200947	200947	200947	200947	T	C	DP=332	SYNONYMOUS_CODING	SILENT	ccT/ccC	P6094
PyYM_07_v1	200983	200983	200983	200983	C	T	DP=317	SYNONYMOUS_CODING	SILENT	aaC/aaT	N6106
PyYM_07_v1	201115	201115	201115	201115	C	T	DP=235	SYNONYMOUS_CODING	SILENT	gtC/gtT	V6150
PyYM_07_v1	201229	201229	201229	201229	A	T	DP=315	SYNONYMOUS_CODING	SILENT	atA/atT	I6188
PyYM_07_v1	201562	201562	201562	201562	G	A	DP=303	SYNONYMOUS_CODING	SILENT	ttG/ttA	L6299
PyYM_07_v1	201657	201657	201657	201657	T	A	DP=236	NON_SYNONYMOUS_CODING	MISSENSE	tTt/tAt	F6331Y
PyYM_07_v1	201826	201826	201826	201826	A	G	DP=313	SYNONYMOUS_CODING	SILENT	ttA/ttG	L6387
PyYM_07_v1	201880	201880	201880	201880	T	C	DP=327	SYNONYMOUS_CODING	SILENT	acT/acC	T6405

PyYM_07_v1	201928	201928	201928	201928	T	C	DP=316	SYNONYMOUS_CODING	SILENT	gaT/gaC	D6421
PyYM_07_v1	202192	202192	202192	202192	G	C	DP=288	SYNONYMOUS_CODING	SILENT	ggG/ggC	G6509
PyYM_07_v1	202327	202327	202327	202327	C	T	DP=314	SYNONYMOUS_CODING	SILENT	taC/taT	Y6554
PyYM_07_v1	202621	202621	202621	202621	C	A	DP=313	SYNONYMOUS_CODING	SILENT	atC/atA	I6652
PyYM_07_v1	202634	202634	202634	202634	T	C	DP=304	NON_SYNONYMOUS_CODING	MISSENSE	Tat/Cat	Y6657H
PyYM_07_v1	202702	202702	202702	202702	T	C	DP=363	SYNONYMOUS_CODING	SILENT	aaT/aaC	N6679
PyYM_07_v1	202954	202954	202954	202954	T	C	DP=292	SYNONYMOUS_CODING	SILENT	gaT/gaC	D6763
PyYM_07_v1	202956	202956	202956	202956	G	A	DP=290	NON_SYNONYMOUS_CODING	MISSENSE	aGc/aAc	S6764N
PyYM_07_v1	203042	203042	203042	203042	A	C	DP=357	NON_SYNONYMOUS_CODING	MISSENSE	Aat/Cat	N6793H
PyYM_07_v1	203048	203048	203048	203048	C	A	DP=359	NON_SYNONYMOUS_CODING	MISSENSE	Cat/Aat	H6795N
PyYM_07_v1	203054	203054	203054	203054	A	C	DP=361	NON_SYNONYMOUS_CODING	MISSENSE	Aat/Cat	N6797H
PyYM_07_v1	203079	203079	203079	203079	A	G	DP=380	NON_SYNONYMOUS_CODING	MISSENSE	aAt/aGt	N6805S
PyYM_07_v1	203101	203101	203101	203101	A	T	DP=397	SYNONYMOUS_CODING	SILENT	atA/atT	I6812
PyYM_07_v1	203240	203240	203240	203240	T	G	DP=396	NON_SYNONYMOUS_CODING	MISSENSE	Ttc/Gtc	F6859V
PyYM_07_v1	203246	203246	203246	203246	T	G	DP=400	NON_SYNONYMOUS_CODING	MISSENSE	Tct/Gct	S6861A
PyYM_07_v1	203285	203285	203285	203285	A	C	DP=415	NON_SYNONYMOUS_CODING	MISSENSE	Aat/Cat	N6874H
PyYM_07_v1	203365	203365	203365	203365	C	T	DP=385	SYNONYMOUS_CODING	SILENT	agC/agT	S6900
PyYM_07_v1	203368	203368	203368	203368	A	G	DP=377	SYNONYMOUS_CODING	SILENT	ttA/ttG	L6901
PyYM_07_v1	203380	203380	203380	203380	T	C	DP=375	SYNONYMOUS_CODING	SILENT	gaT/gaC	D6905
PyYM_07_v1	203468	203468	203468	203468	G	C	DP=285	NON_SYNONYMOUS_CODING	MISSENSE	Gaa/Caa	E6935Q
PyYM_07_v1	203469	203469	203469	203469	A	C	DP=283	NON_SYNONYMOUS_CODING	MISSENSE	gAa/gCa	E6935A
PyYM_07_v1	203472	203472	203472	203472	C	A	DP=283	NON_SYNONYMOUS_CODING	MISSENSE	gCg/gAg	A6936E
PyYM_07_v1	203512	203512	203512	203512	T	C	DP=237	SYNONYMOUS_CODING	SILENT	atT/atC	I6949
PyYM_07_v1	203788	203788	203788	203788	C	T	DP=314	SYNONYMOUS_CODING	SILENT	aaC/aaT	N7041
PyYM_07_v1	203884	203884	203884	203884	G	A	DP=321	SYNONYMOUS_CODING	SILENT	caG/caA	Q7073
PyYM_07_v1	204316	204316	204316	204316	T	C	DP=296	SYNONYMOUS_CODING	SILENT	aaT/aaC	N7217
PyYM_07_v1	204328	204328	204328	204328	C	T	DP=309	SYNONYMOUS_CODING	SILENT	ccC/ccT	P7221
PyYM_07_v1	204775	204775	204775	204775	T	C	DP=357	SYNONYMOUS_CODING	SILENT	gaT/gaC	D7370
PyYM_07_v1	205096	205096	205096	205096	T	C	DP=303	SYNONYMOUS_CODING	SILENT	tcT/tcC	S7477

PosiBon, nucleoBde posiBons on chromsome 7; Depth, sequence read coverage; the numbers indicate reads covering the site; Coding_synonymous_nonsynonymous, the nature of nucleoBde subsBtuBons; MutaBon, mutaBon types. The *Plasmodium yoelii yoelii* YM sequence was from public database (hQps://www.sanger.ac.uk/resources/downloads/protozoa/plasmodium-yoelii.html), and the *Plasmodium y. nigeriensis* was from our own sequencing as described (Niar et al., Mol Biochem Parasitol 194, 9-15). The changes leading to amino acid changes are color-highlighted.

Supplementary Table 5. Sequences of primers used for PCR and for building plasmid constructs.

Primers for amplifying cDNA tiling thePlasmodium yoelii HECT E3 uniquitine ligase gene (5'-3')

Name	Forward primer sequences (5'-3')	Name	Reverse primer sequences (5'-3')	Product length (bp)
p1	ATGAAAATCCAACTTCCCAAAG	p2	CAATTAATGTATCTGTTGGGGA	2737
p3	TCCCCAACAGATACATTAATTG	p4	TTTCTATCAATAAAAGGTCCAG	1060
p5	CTGGACCTTTTATTGATAGAAA	p6	GAACTAGAGTTGTAAGAATCAGA	1815
p7	TCTGATTCTTACAACTCTAGTTC	p8	ATTTCGTCGGTTTCTATTATGA	1744
p9	TCATAATAGAAACCGACGAAAT	p10	CAATTTGTGCATACTTCTGTAT	1700
p11	ATACAGAAGTATGCACAAATTG	p12	ATATGTGTTTATGGAATCTGGA	2653
p13	TCCAGATTCCATAAACACATAT	p14	TTCAGTATCTTCTTCTTCGT	1410
p15	ACGAAGAAGAAGATACTGAAGA	p16	TCGTCATTTCCCTCAGATATA	1532
p17	TATATCTGAGGGAAATGACGA	p18	AGCTGATTCTTCTTCATTTAGT	1489
p19	ACTAAATGAAGAAGAATCAGC	p20	CGTTTAATCGAGTATCTTCTGT	2926
p21	ACAGAAGATACTCGATTAAACG	p22	GAACTAGACTCTGCTGAATTAT	1171
p23	ATAATTCAGCAGAGTCTAGTTC	p24	TTATATAAATCCGAAACCCTCT	2263

Primers for linear *Pyhebl* knockout construct (5'-3')

P1F1	GCTAAGGCTATTTTTATATTTTC	P1R1	GCTGGGCTGCAGGAA TTATCAAGCTTTACATTATCACGTCCTTAAA
P1F2	TTTAAGGACGTGATAATGTAAAGCTTGATAATTCCTGCAGCCCAGC	P1R2	CAAATGATGTTTTTTCCTTCAATT TCGGTAC ATATACCCATACATTAAGT
P1F3	ACTTAATGTATGGGTATATGTACCGAAATTGAAGGAAAAAACATCATTTG	P1R3	ATGATGGTGATGTTGGTGAA
Pl1	CTCTTCAATGATTCATAAATAGTTGGAC	Pl3	ATGATGGTGATGTTGGTGAA
Pl2	TATGTTGTCTCTTTCAATGATTC	Pl4	TGGTGATGTTGGTGAAGAAA

Primers insertion at 5' UTR of the *Pyhebl* gene (5'-3')

PiF1	CGGGTACCTGCAACATATTTCCAAAGCAT	PiR1	AGGCGCCGTACATAAGCTATGAAAGTGTT
Pi1	TGTTGAATTAGATGGTGATGT	Pi2	AGAGGCATTTATTTTGTGGT
Pi3	CGAAGAATGAGAATGAAATAGCG	Pi4	CTTAACTATGCGGCATCAGA
Pi5	GATTCATAAATAGTTGGACTTGA	Pi6	CATTGCCATTATTAACTTCTTCA

Primers for 5 UTR insertion at 18S rRNA gene (5'-3')

18SF1	TTGAAATTAGCAGCATAGCA	18SR1	CTTAACTATGCGGCATCAGA
18SF2	CTGAAGTCAAGTTTGAAGGT	18SR2	AAGACATCCATCGTTAAGATT

Primers for vector constructions using CRISPR/Cas9 system and for checking transfectants

Primer name	Primer sequence (5'-3')	Targets and descriptions
HEUL-1K55	atcgAAGCTTCAACAAAATTCCTATACATTACTATTC	Left arm, 5'UTR
HEUL-1K53	gcatCCATGGTGTTTACTCATAATTTAGAAAATAATAAAACA	Left arm, 5'UTR
HEUL-1K35	gcatCTCGAGATAATTCGAATCTTCTATGTATATATATTACC	Right arm, 3'UTR
HEUL-1K33	gcatGAATTCAATAATGGAAGAACAATAATTCTAC	Right arm, 3'UTR
K1-sgRNA-F	TATTGGATGTAAAAAATTAGGAAT	KO targeting sgRNA
K1-sgRNA-R	AAACATTCCTAATTTTTTACATCC	KO targeting sgRNA
K1-F1	AAACAACTTTTATCATTAAGAAAAATTTATAG	5'UTR
K1-R1	GTGAGTTTCTCATACGAAATAGTG	3'UTR
K1-F2	GATGTACTTCAAAATGCTTATGAT	Coding region
K1-R2	CATTATATGGTTCTATAGGGT	Coding region
HEUL-2K55	atcgAAGCTTGATGTACTTCAAAATGCTTATGAT	Left arm, coding region
HEUL-2K53	gcatCCATGGCATTATATGGTTCTATAGGGT	Left arm, coding region
HEUL-2K35	gcatCTCGAGTTTTAATTGCTGCATATGCAGA	Right arm, coding region
HEUL-2K33	gcatGAATTCATTTTCAGTTGTTTGTACATGTGA	Right arm, coding region
K2-sgRNA-F	TATTGGTGATATAGCAGTTTCAAT	KO targeting sgRNA
K2-sgRNA-R	AAACATTGAAACTGCTATATCACC	KO targeting sgRNA
K2-F1	TACATCTATATTTCCAAACCAAGTT	Coding region
K2-R1	CATTCTCATTTATGTTGCCTTGAT	Coding region
K2-F2	GATATTAATATGAACATATCAGAAGA	Coding region
K2-R2	CGATTGAAACTGCTATATCACCA	Coding region
HEUL-R1F	gcatCCATGGAATCGAGATCAGAAACTAACCA	Homology arm for R1
HEUL-R1R	gcatCTCGAGGTATCTGTTGGGGAATACC	Homology arm for R1
HEUL-R1LF	TGGAACCATCTCC**G**ATCTTGACAATAGCACAACAAAATACTTTAAAGAC	Synonymous mutations introduced in R1 sgRNA binding site
HEUL-R1LR	TTGTTGTGCTATTGTCAAGATCGGAGATGGTTCCATTATACATGTATGA	Synonymous mutations introduced in R1 sgRNA binding site
R1-sgRNA-F	TATTGCTATTGTTAAAATAGGAGA	R1 targeting sgRNA
R1-sgRNA-R	AAACTCTCCTATTTTAACAATAGC	R1 targeting sgRNA
HEUL-R2F	gcatCCATGGCTCCGATTTTACAAGTTACATACCT	Homology arm for R2
HEUL-R2R	gcatCTCGAGGAATCAAAATCACCAAGTAAAGT	Homology arm for R2
HEUL-R2LF	AATGTTACACCCGTTCACGAGAATTAATCAAACATATGGCAA	Synonymous mutations introduced in R2 sgRNA binding site
HEUL-R2LR	CATATGTTTGATTAATTCTCGTGAACGGGTGTAACATTTTTGAAGT	Synonymous mutations introduced in R2 sgRNA binding site
R2-sgRNA-F	TATTGTTTGATTAATTCTTGTAAA	R2 targeting sgRNA
R2-sgRNA-R	AAACTTTACAAGAATTAATCAAAC	R2 targeting sgRNA
HM3-F	ACGCTAATCGTAGCTAGCCTGCT	Introduced sense primer for integration detection
HM5-R	CGATTAGCGTCCGCGGGGACCAT	Introduced reverse primer for integration detection

Primers for amplifctiton of tubulin alpha

Primer name	Primer sequence (5'-3')
tub_af1	ATGAGAGAAGTAATAAGTATACATG
tub_ar1	TTAATAATCTGCTTCATATCCTTCGTC

Note: Nucleotides in red are restriction sites, and those in green are substitions introduced into gRNA to prevent cleavage by the enzyme.

Supplementary Table 6. Day 4 parasitemia of different R1 replacement clones after injecting BALB/c mice with 1×10^6 parasites (*i.v.*)

Parasite	Mouse number	Day 4 parasitemia	Averaged parasitemia	Standard deviation	Standard error	Averaged parasitemia (combined)	Standard deviation (combined)	Standard error (combined)
17XL	1	89.35%						
	2	89.28%						
	3	82.88%						
	4	71.07%	83.15%	8.60%	4.30%			
Repeat	5	80.58%						
	6	79.76%						
	7	87.14%						
	8	77.53%	81.25%	4.13%	2.07%	82.20%	6.33%	2.24%
N67	1	48.61%						
	2	44.54%						
	3	39.87%						
	4	46.22%	44.81%	3.69%	1.85%			
Repeat	5	41.88%						
	6	46.04%						
	7	44.73%						
	8	44.04%	44.17%	1.74%	0.87%	44.49%	2.69%	0.95%
17XL-R1$^{17XL-112}$	1	76.59%						
	2	92.17%						
	3	93.88%						
	4	94.44%	89.27%	8.51%	4.25%			
Repeat	5	93.66%						
	6	94.90%						
	7	73.83%						
	8	96.78%	89.79%	10.72%	5.36%	89.53%	8.96%	3.17%
17XL-R1$^{17XL-132}$	1	6.26%						
	2	10.21%						
	3	10.50%						
	4	13.05%	10.01%	2.80%	1.40%			
N67-R1$^{17XL-234}$	1	56.60%						
	2	54.27%						
	3	55.49%						
	4	57.74%	56.02%	1.49%	0.74%			
Repeat	5	42.40%						
	6	47.83%						
	7	56.68%						
	8	49.21%	49.03%	5.88%	2.94%	52.53%	5.46%	1.93%
N67-R1$^{17XL-213}$	1	34.50%						
	2	37.02%						
	3	35.38%						
	4	47.51%	38.60%	6.03%	3.02%			
17XL-R1$^{N67-3323}$	1	89.49%						
	2	83.74%						
	3	84.15%						
	4	91.88%	87.32%	4.01%	2.01%			
Repeat	5	91.88%						
	6	86.59%						
	7	83.85%						
	8	85.32%	86.91%	3.50%	1.75%	87.11%	3.49%	1.24%
17XL-R1$^{N67-3334}$	1	63.88%						
	2	76.72%						
	3	57.07%						
	4	57.53%						
	5	61.56%	63.35%	7.99%	3.57%			
Repeat	6	68.80%						
	7	75.18%						
	8	83.85%						
	9	61.17%						
	10	80.31%	73.86%	9.08%	4.06%	68.61%	9.78%	3.09%
N67-R1$^{N67-421}$	1	44.68%						
	2	48.39%						
	3	42.35%						
	4	49.71%	46.28%	3.38%	1.69%			
Repeat	5	41.99%						
	6	51.94%						
	7	44.23%						
	8	44.90%	45.77%	3.59%	1.79%	46.02%	3.59%	1.27%
N67-R1$^{N67-414}$	1	17.57%						
	2	14.36%						
	3	11.77%						
	4	15.41%	14.78%	2.41%	1.20%			

Note: Those highlighted in orange color are groups with unexpected growth phenotypes, deviating greatly from the parents.
Repeats are infections of mice with the same parasite clones at different times, usually several weeks or motnhs apart.

Supplementary Table 7. Parasitemia of mice infected with R2 allelic exchanged parasites.

Mouse group	Mouse number	Parasite clone	Day 4 parasitemia
1	1	17XL	89.35%
	2		89.28%
	3		82.88%
	4		71.07%
		Average	83.15%
		SD	8.60%
		SE	4.30%
2	1	N67	48.61%
	2		44.54%
	3		39.87%
	4		46.22%
		Average	44.81%
		SD	3.69%
		SE	1.85%
3	1	N67-R2$^{17XL-11}$	45.04%
	2		29.45%
	3		51.41%
	4		46.63%
		Average	43.13%
		SD	9.52%
		SE	4.76%
4	1	N67-R2$^{17XL-12}$	52.78%
	2		49.76%
	3		43.78%
	4		53.13%
		Average	49.86%
		SD	4.33%
		SE	2.16%
5	1	N67-R2$^{17XL-23}$	24.70%
	2		43.90%
	3		53.64%
	4		54.19%
		Average	44.11%
		SD	13.78%
		SE	6.89%
6	1	N67-R2$^{17XL-32}$	61.01%
	2		56.76%
	3		58.69%
	4		48.72%
		Average	56.30%
		SD	5.34%
		SE	2.67%
7	1	N67-R2$^{17XL-33}$	56.22%
	2		58.03%
	3		51.38%
	4		54.00%
		Average	54.91%
		SD	2.87%
		SE	1.44%
8	1	N67-R2$^{17XL-43}$	54.96%
	2		54.57%
	3		34.83%
	4		48.69%
		Average	48.26%

		SD	9.40%
		SE	4.70%
9	1	N67-R2[17XL-52]	55.60%
	2		56.59%
	3		53.59%
	4		36.43%
		Average	50.55%
		SD	9.50%
		SE	4.75%
10	1	N67-R2[17XL-54]	42.16%
	2		49.95%
	3		51.08%
	4		47.69%
		Average	47.72%
		SD	3.97%
		SE	1.98%

Second set of experiemts

11	1	N67-R2[17XL-33]	42.37%
	2		51.23%
	3		57.10%
	4		53.17%
		Average	50.96%
		SD	6.23%
		SE	3.12%
12	1	N67-R2[17XL-52]	51.67%
	2		55.75%
	3		53.65%
	4		58.48%
		Average	54.89%
		SD	2.92%
		SE	1.46%
13	1	N67-R2[N67-22]	47.57%
	2		44.83%
	3		48.78%
	4		41.67%
			33.60%
		Average	43.29%
		SD	6.07%
		SE	3.04%
14	1	N67-R2[N67-53]	40.79%
	2		53.84%
	3		43.02%
	4		43.96%
		Average	45.40%
		SD	5.78%
		SE	2.89%

Note: Number in red are averaged parasitemia and standard error (SE). 1×10^6 infected red blood cells were injected into BALB/c mice (*i.v.*). Repeat infections were performed approximately four months later. N67-R2[N67] parasites were not available for the first experiments. The numbers highlighted in yellow are "outliers" likely due to technical variation during injection of mice, which causes the relateively large SEs.

References

1 Sen, S. & Churchill, G. A. A statistical framework for quantitative trait mapping. *Genetics* **159**, 371-387 (2001).

2 Li, J. *et al.* Linkage maps from multiple genetic crosses and loci linked to growth-related virulent phenotype in Plasmodium yoelii. *Proc Natl Acad Sci U S A* **108**, E374-382, doi:10.1073/pnas.1102261108 (2011).

3 Ecker, A., Moon, R., Sinden, R. E. & Billker, O. Generation of gene targeting constructs for Plasmodium berghei by a PCR-based method amenable to high throughput applications. *Mol Biochem Parasitol* **145**, 265-268 (2006).

4 Qi, Y. *et al.* Regulation of Plasmodium yoelii oocyst development by strain- and stage-specific small-subunit rRNA. *MBio* **6**, e00117, doi:10.1128/mBio.00117-15 (2015).

5 Zhang, C. *et al.* Efficient editing of malaria parasite genome using the CRISPR/Cas9 system. *MBio* **5**, e01414-01414, doi:10.1128/mBio.01414-14 (2014).
